# Lubaniski on the Urine of Pregnant Women

**Published:** 1842-12-24

**Authors:** 


### ANALECTA.

*Lubaniski on the Urine of Pregnant Women.*—It is seldom as acid as in other individuals, occasionally it is neutral, and sometimes alkaline, and generally light-colored. Donné suspected that the salts of lime for the most part are diminished during pregancy, and that a part of them is taken to supply the materials for the formation of the fœtal bone; and he found in many experiments instituted for this purpose, that by the addition of thirty parts of hydro-chloride of lime to fifty parts of urine, there was a precipitate of from forty to fifty parts of salt of lime in common urine, whereas in that of pregnancy, the most he ever detected was thirty, and very often not near so much. Before making the experiment, the urine to be tried must be tested, to ascertain if it be alkaline or acid, and if acid, a few drops of ammonia must be added to render it alkaline, since the precipitate from phosphate of lime is soluble in weak acids. If the experiment be made with solution of baryta, there will be in healthy urine a precipitate of from twelve to fifteen parts of salts of baryta; in the pregnant from five to eight, after twelve hours' rest. With reference to the question of pregnancy, Donné has, out of thirty-six cases, only twice been deceived. Lubaniski found it decisive in three cases of pregnancy, where the manual examination and auscultation proved unavailing. He proposes the following questions for investigation. 1. At wnat period of pregnancy does this diminution of the salts of lime take place? 2. Is it always constant? 3. In what relation does it stand to the increase of fœtal ossification? 4. At what period does it cease?—*Dublin Journal of Med. Sci., Nov.* 1842, *from Ann. d' Obstetrique des Maladies des Femmes et des Enfans.*

